# Assessment of Chemical, Physico-Chemical and Sensory Properties of Low-Sodium Beef Burgers Formulated with Flours from Different Mushroom Types

**DOI:** 10.3390/foods12193591

**Published:** 2023-09-27

**Authors:** Carmen Botella-Martínez, Nuria Muñoz-Tebar, Raquel Lucas-González, José A. Pérez-Álvarez, Juana Fernández-López, Manuel Viuda-Martos

**Affiliations:** IPOA Research Group, Centro de Investigación e Innovación Agroalimentaria y Agroambiental (CIAGRO), Miguel Hernández University, 03312 Alicante, Spain; c.botella@umh.es (C.B.-M.); nmunoz@umh.es (N.M.-T.); raquel.lucasg@umh.es (R.L.-G.); ja.perez@umh.es (J.A.P.-Á.); j.fernandez@umh.es (J.F.-L.)

**Keywords:** button mushroom, oyster mushroom, portobello mushroom, low-salt, reformulation

## Abstract

It is now widely demonstrated that excessive salt consumption can cause various health problems, and meat products are among the foods most consumed with a high salt content. For that, the aim of this work was to assess the effects of the utilization of flours obtained from oyster mushrooms (*Pleurotus ostreatus*), button mushrooms (*Agaricus bisporus*), and portobello mushrooms (*Agaricus brunnescen*) as salt replacers on chemical, physicochemical, and sensory properties of beef burgers. The fat and protein content was not affected by the inclusion of mushroom flour, while the sodium content was reduced by 55–61% compared to the control sample. The control sample had the lowest values for cooking loss and shrinkage (12.29 and 18.69%, respectively) whilst the reformulated samples had higher values ranging between 16.08 and 18.88% for cooking loss, respectively, and between 19.55 and 28.25% for shrinkage, respectively. The reformulated samples showed higher lipid oxidation values (ranging from 0.18 and 0.20 mg malondialdehyde/kg sample) than the control sample. Sensorially, all parameters analyzed were not affected by the replacement of sodium chloride by the different mushroom flours. The use of flours obtained from different mushroom flours is a viable alternative to be used as sodium chloride replacers in the preparation of beef burgers.

## 1. Introduction

Nowadays, it has been widely recognized that a high intake of sodium chloride may be responsible for several illnesses, including cardiovascular diseases, kidney failure, and several types of cancer [1]. In this sense, it has been estimated that the global mean intake of sodium chloride is about 9–12 g/day, representing twice the maximum recommended intake level in adults (5 g sodium chloride/day equivalent to 2 g sodium/day) [2]. In response to this concern, the purpose of reducing individual sodium chloride consumption by 30% by 2025 was established in line with global health goals [3]. Against this backdrop, a remarkable number of health organizations as well as government policies, have launched campaigns to reduce the sodium chloride content in food, encouraging the food industry, which is fully aware of the problem, to focus on the reformulation of products with low sodium chloride content. Nevertheless, it should be borne in mind that sodium chloride is an essential nutrient for the organism with several functions, including nerve impulse transmission, myokinesis, absorption of nutrients in the intestine, and control of hormones, among others [4]. On the other hand, sodium chloride has several roles when added to food products. First and foremost, sodium chloride provides a salty taste to foods and improves the effect of other flavor components [5]. Secondly, sodium chloride has specific properties, including protein solubility, control of fermentation rate, improvement of product texture, and control of enzymatic reactions, among others, that are essential for the physical and technological final product characteristics [6]. Finally, sodium chloride exerts an antimicrobial effect due to its ability to reduce water activity [7]. For all these reasons, the elimination of sodium chloride in food products is a complicated task. 

The food industry is implementing several strategies to reduce the salt content of food products, including the replacement of sodium chloride with flours or extracts obtained from edible mushrooms [8,9]. A very interesting feature of edible mushrooms is their content of umami-like flavorings, such as the free glutamic and aspartic amino acids and 5′-ribonucleotides, for which flavor-stimulating properties have also been described [10]. Different peptides (glutamic-containing) with unique flavoring properties have also been found in mushrooms and may even interact with other volatile compounds, influencing the final aroma and flavor of foods [11]. This flavoring property means that mushrooms can be used in meat products as a substitute for salt. Additionally, mushrooms could be considered a potential ingredient in the development of low-sodium food products because it is possible to find several bioactive compounds, including proteins, dietary fiber, polyphenolic compounds, vitamins, and minerals in their composition [12,13]. It is also important to note that due to the dietary fiber content of mushrooms, numerous techno-functional properties of the products to which they are added can be improved, including water or oil retention capacity, gelling capacity, and a reduction in cooking losses.

In the scientific literature, it is possible to find several works where the substitution of sodium chloride with edible mushrooms is determined. Such is the case of the study conducted by França et al. [14] in which the addition of umami ingredient from shiitake (*Lentinula edodes*) stipes in low-sodium beef burgers was evaluated. They noticed a slight increase in lightness and yellowness without affecting redness, pH, and water activity. Cooking loss and diameter reduction did not decrease, and an increase in proline and phenylalanine was detected in burgers enriched with umami ingredients. In another study, Cerón-Guevara et al. [15] formulated beef patties with *Agaricus bisporus* and *Pleurotus ostreatus* flours (2.5 and 5.0%) as salt and fat replacers with acceptable sensory characteristics and no effect on hardness, gumminess, and chewiness compared to control samples. In general terms, when mushrooms are used as salt replacers in meat products, the way to add them is to employ an aqueous extract or fresh mushroom flour with a very low degree of salt replacement. Therefore, the aim of this work was to assess the effects of the utilization of flours obtained from three edible mushrooms, namely oyster mushroom (*P. ostreatus*), button mushroom (*A. bisporus*), and portobello mushroom (*Agaricus brunnescen*) as sodium chloride replacers (at 75%) on the chemical, physico-chemical, and sensory properties of a beef burger. 

## 2. Materials and Methods

### 2.1. Mushroom Flour

**Figure 1:**
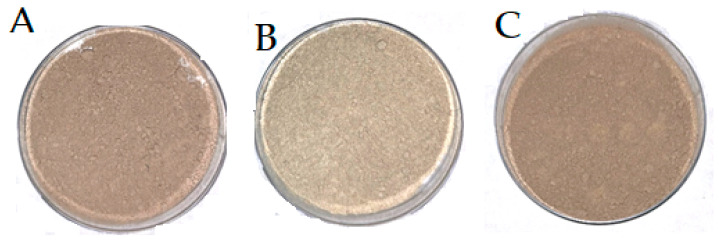
Mushroom flour obtained from (**A**) *Agaricus brunnescens*, (**B**) *Pleutorus ostreatus,* and (**C**) *Agaricus bisporus*.

Oyster mushrooms (*Pleurotus ostreatus*), button mushrooms (*Agaricus bisporus*), and portobello mushrooms (*Agaricus brunnescen*) were purchased from a local supermarket in Orihuela, Spain. Samples were wiped with a wet cloth to remove substrate residues and soil. Then, the samples were cut into 1 × 1 × 1 cm pieces and were dried at 45 °C for 24 h. After that, dehydrated samples were ground in a Hammer Mill M20 (IKA, Staufen, Germany) and passed through a sieve (particle size < 0.417 mm) to obtain the flour. The three fractions obtained (Figure 1) were: oyster mushroom (OM) flour (moisture 4.20%, dietary fiber 46.5%, lipids 1.90%, protein 17.48%, and ash 5.90%), button mushroom (BM) flour (moisture 4.18%, dietary fiber 30.0%, lipids 2.34%, protein 20.12 %, and ash 11.78%), and portobello mushroom (PM) flour (moisture 4.41%, dietary fiber 32.10%, lipids 1.95%, protein 18.23%, and ash 10.23%). 

### 2.2. Elaboration of Low-Sodium Beef Burgers 

To elaborate the burgers (twenty for each formulation), the beef round muscle (moisture 72.20%, lipids 3.55%, protein, 23.51%, and ash 0.74%), and pork backfat (moisture 11.23%, lipids 73.42%, proteins 12.79% protein, and ash 0.56%) were ground in a mincer (Olotinox, Olox, Spain) using a 10-mm plate. After grinding, the ingredients were mixed manually for 10 min in the following order: beef burger, pork backfat, sodium chloride, water, and pepper. This original formula was used as a control sample (CS), while in the other three formulations, sodium chloride (75%) was substituted by the different mushroom flours, as shown in Table 1.

**Figure 2:**
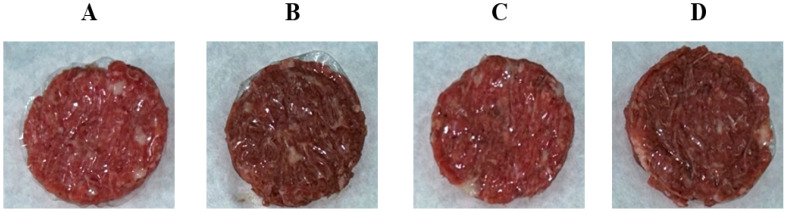
(**A**) Raw control sample; (**B**) raw low-sodium chloride beef burgers where *Agaricus brunnescens* flour was used as sodium chloride replacer; (**C**) raw low-sodium chloride beef burgers where *Pleutorus ostreatus* flour was used as sodium chloride replacers; (**D**) raw low-sodium chloride beef burgers where *Agaricus bisporus* flour was used as sodium chloride replacer. Button mushroom flour and portobello mushroom flour were used as partial (75%) sodium chloride replacers.

After obtaining the meat batter, 45 g portions were weighed and shaped (6 cm diameter and 1 cm thick) using a commercial burger maker. Plastic packaging film was used to help maintain the shape of the patties previously to be packed into PVC hermetic bags and stored at 4 °C until analysis (Figure 2).

### 2.3. Chemical Composition

The chemical composition (moisture, protein, fat, and ash content) of the raw and cooked low-sodium chloride beef burgers was assessed following the methodology described by AOAC [16]. The mineral content was determined using inductively coupled plasma mass spectrometry (ICP-MS) Shimadzu MS-2030 (Shimadzu, Kyoto, Japan). The standard compounds were diluted and utilized to calibrate the ICP-MS for mineral analysis in lyophilized beef samples. ICP-MS operated with the following conditions: carrier gas 0.70 L/min; plasma gas 9.0 L/min; auxiliary gas 1.10 L/min; radio frequency 1.2 kW, energy filter 7.0 V.

### 2.4. Physico-Chemical Properties

#### 2.4.1. pH

The pH of the raw low-sodium chloride beef burgers was measured using a portable pH-meter (Crison micro pH-meter 2001 model GLP 21, Crison Instrument S.A., Barcelona, Spain) equipped with a penetrating electrode.

#### 2.4.2. Water Activity

The water activity analysis for the raw beef burgers was carried out using a water activity analyzer Novasina TH-500 (Novasina, Axair Ltd., Pfaeffikon, Switzerland) at 25 °C. 

#### 2.4.3. Color Analysis

The instrumental color of low-sodium chloride beef burgers was determined on the surface of raw and cooked beef burgers using a Minolta spectrophotometer Model CM-700, with the following specifications: D_65_ illuminating source, 10° observer angle and 11-mm aperture (Konica Minolta Sensing, Inc., Tokyo, Japan). The lightness (L*), redness (a*), and yellowness (b*) were determined following CIEL*a*b* color coordinates. Additionally, the reflectance spectra (360–740 nm) were recorded.

Total color differences (ΔE) of each reformulated sample with respect to the control sample were determined with the following Equation (1).

(1) $$\Delta E=\sqrt{{\left({\Delta L}^{*}\right)}^{2}+{\left({\Delta a}^{*}\right)}^{2}+{\left({\Delta b}^{*}\right)}^{2}}$$

The differences in ΔE were divided into 6 points based on the color difference classification by the National Bureau of Standards (Equation (2)):

NBS = ΔE × 0.92 (2)

The total color differences were classified as trace (NBS 0–0.5), slight (NBS 0.5–1.5), noticeable (NBS 1.5–3.0), appreciable (NBS 3.0–6.0), much (NBS 6.0–12.0), and very much (NBS > 12) according to Jeong et al., [17]

Additionally, the redness index (RI) was calculated following the recommendations of the American Meat Science Association [18] using the below Equation (3)

(3) $$RI=\frac{a^{*}}{b^{*}}$$

#### 2.4.4. Texture Analysis

Texture profile analysis was realized in cooked low-sodium chloride beef burgers in a TA-XT2i Texture Analyzer (Stable Micro Systems, Surrey, UK). Pieces of 2 cm × 2 cm × 2 cm of each formulation were submitted to two-cycle compression to 75% at room temperature and a constant velocity of 1 mm/s. The parameters determined were hardness (N), springiness (mm), cohesiveness, and chewiness (N × mm) [19].

### 2.5. Cooking Properties

The cooking loss (percentage of weight loss after cooking) and the diameter reduction (% shrinkage) of cooked low-sodium chloride beef burgers were calculated using Equations (4) and (5), respectively.

(4) $$\mathrm{Cooking}\;\mathrm{loss}\;\left(\%\right)=\left(\frac{Weight\;raw\;burger-Weight\;cooked\;burger}{Weight\;raw\;burger}\right)\times 100$$

(5) $$\mathrm{Shrinkage}\;\left(\%\right)=\left(\frac{Raw\;diameter-Cooked\;diameter}{Raw\;diameter}\right)\times 100$$

### 2.6. Lipid Oxidation

Lipid oxidation values of cooked low-sodium chloride beef burgers were assessed following the TBARs (thiobarbituric acid reactive substances) methodology described by Sobral et al. [20]. The results were expressed as mg malondialdehyde (MDA)/kg sample. 

### 2.7. Sensory Evaluation

Sensory analysis was performed on 75 untrained panelists recruited from the staff and students of Miguel Hernandez University. For that, a nine-point hedonic scale was used, and the panelists were asked to score the cooked low-sodium chloride beef burgers from 1 (dislike extremely) to 9 (like extremely) on seven attributes: visual aspect, global color, hardness, global flavor, salty taste, rancidity, as well as general acceptability. 

### 2.8. Statistical Analysis

The full process (burger manufacture and all analysis) was replicated three times (three independent batches). Each repetition was realized on a different manufacturing day, and each batch was assessed in triplicate. When variables followed a normal distribution, the data obtained were analyzed by one-way ANOVA, and the Tukey post-hoc test was performed at a 95% significance level using the statistical package SPSS v. 24 for Windows (SPSS INC., Chicago, IL, USA). 

## 3. Results and Discussion

### 3.1. Chemical Composition

Table 2 shows the values obtained for moisture, proteins, fats, and ash of the raw and cooked low-sodium chloride beef burgers where oyster mushroom flour, button mushroom flour, and portobello mushroom flour were used as partial (75%) sodium chloride replacers. In raw burgers, the replacement of sodium chloride by edible mushroom flours had no significant effect (*p* > 0.05) on moisture content with respect to the control sample. However, the ash content was profoundly affected (*p* < 0.05) by the replacement of sodium chloride with different mushroom flours. 

Thus, a reduction in this parameter was achieved without significant differences (*p* > 0.05) between the mushroom flours used. These results were in concordance with those reported by Cerón-Guevara et al. [15], who reported a reduction in the ash content of beef patties added with flours obtained from *Agaricus bisporus* and *Pleurotus ostreatus*. On the other hand, the addition of different edible mushroom flours, such as sodium chloride replacer, increased the protein content (*p* < 0.05) in reference to control samples. Thus, BPM showed the highest values, followed by BBM and BOM, without significant differences (*p* > 0.05) between them. For fat content, the use of mushroom flours did not cause significant changes (*p* > 0.05) between the control sample and the BBM and BPM samples. However, the BOM sample showed the lowest values (*p* < 0.05). In cooked burgers, the control sample had the highest values (*p* < 0.05) for moisture and ash content. In the case of moisture, this could be explained by the fact that sodium chloride allows the solubilization of myofibrillar proteins, implying a higher water retention capacity in meat batter and hence these higher values [21]. The reduction in the ash content of reformulated burgers might be due to a reduction in the sodium chloride content. The fat content was not affected (*p* > 0.05) by the replacement of sodium chloride, whereas the protein content increased in reformulated samples (*p* < 0.05) with respect to the control, without differences (*p* > 0.05) between them. This increase in protein content was related to the addition of different edible mushroom flours, which contain around 19–37% of protein [22].

As regards the mineral content, in the raw samples (Table 2), as expected, the control sample showed the highest values (*p* < 0.05), while in the reformulated samples, a reduction in the sodium content of 63.33, 54.83, and 56.18% was obtained for the BBM, BOM, and BPM samples, respectively with respect to the control. With these values obtained, according to European regulations [23], BBM, BOM, and BPM burgers might be labeled as “reduced in sodium” since they reached a reduction of more than 25%. This reduction in sodium content agrees with those reported by Patinho et al. [8] in beef burgers added with *A. bisporus* flour. Except for calcium, for the rest of the minerals analyzed (potassium, iron, magnesium, and zinc), the replacement of sodium chloride with the different edible mushroom flours led to an increase in the content of these minerals (*p* < 0.05) compared to the control. In reference to the sodium content of cooked samples, the same behavior was achieved. The sodium reduction (*p* < 0.05), in relation to the control sample, was 55.21, 54.97, and 61.02% for the BBM, BOM, and BPM samples, respectively. It is important to highlight that the replacement of sodium chloride with the different mushroom flours in cooked burgers provides 400.61–459.60 mg sodium/100 g burger, which corresponds to around 20–23% of the recommended daily amount of sodium (2 g sodium/day) established by the World Health Organization [24].

### 3.2. Physico-Chemical Properties

All the physico-chemical properties of raw low-sodium chloride beef burgers where oyster mushroom flour, button mushroom flour, and portobello mushroom flour were used as partial (75%) sodium chloride replacers are given in Table 3. Regarding pH values, the replacement of sodium chloride by BBM and BPM produced a slight increase in pH values with respect to the control sample (*p* < 0.05). 

However, no statistical differences were found (*p* > 0.05) between the CS and burgers with BOM. This increase in pH values may be explained by the existence of basic amino acids, including histidine, lysine, and arginine, in the button mushroom and portobello mushroom flours [15,25]. This increase in pH values agreed with the values reported by França et al. [14] in low-salt beef burgers added with shiitake by-products or Patinho et al. [8] in beef burgers added with button mushroom by-products.

In reference to water activity (Table 3), the replacement of sodium chloride by edible mushroom flours produces an increase (*p* < 0.05) in this parameter with respect to the control sample. This fact occurs due to that the addition of sodium chloride might reduce the vapor pressure of the food product, thereby reducing water activity [26]. Thus, the samples with lower sodium chloride content showed higher water activity. The results obtained in this work corroborate those reported by França et al. [14] in beef burgers with salt reduction added with shiitake by-products. 

When fresh mushroom or mushroom flour is added to foods, a modification in color parameters is unavoidable. In this case, lightness (L*) and yellow-blue (b*) coordinates were not statistically different (*p* > 0.05) between the control sample and the samples in which mushroom flours were used as sodium chloride substitutes (Table 3). However, the red-green (a*) coordinate was deeply affected by the substitution of sodium chloride by the mushroom flours. Thus, the CS had the highest values (*p* < 0.05) whilst no differences were found (*p* > 0.05) between the BBM, BOM, and BPM samples. This variation in the a* coordinate is clearly seen in the redness index, which relates the a* and b* coordinates. Thus, the samples in which the sodium chloride was replaced by the different mushroom flours showed much lower values (*p* < 0.05) with respect to the control sample. This reduction in a* coordinate and in the redness index is a significant result because the red color of meat products is one of the main characteristics evaluated by the consumer for high sensory acceptance [27].

The analysis of reflectance spectra of meat products in general and burgers, in particular, might be helpful to understanding the later changes in these products due to the application of new ingredients comparing the pattern and spectral intensity changes of one spectrum with those of other spectrums. Figure S1 shows the reflectance spectra (360–740 nm) achieved for the raw low-sodium chloride beef burgers where oyster mushroom flour, button mushroom flour, and portobello mushroom flour were used as partial sodium chloride replacers. The spectral analysis of the samples showed that the reflectance spectrum of all the samples presented a reflectance spectrum corresponding to the oxymyoglobin state according to the American Meat Science Association Guidelines for Meat Color Determination [28]. 

The use of the different mushroom flours as sodium chloride replacer only affected % reflectance in BOM samples, increasing these values (*p* < 0.05) in all the wavelengths analyzed except in the wavelengths of 360 nm where all the samples presented an isosbestic point (*p* > 0.05). The statistical analysis applied to the different wavelengths showed that there were no significant differences (*p* > 0.05), which implies that there were isosbestic points between the wavelengths 370 and 470 nm for the CS, BBM, and BPM samples. For wavelengths compressed between 480 and 510 nm, no significant differences (*p* > 0.05) were found between the CS, BBM, and BPM samples. At 530 nm, isosbestic points were present only for BBM and BPM samples. From wavelengths between 540 and 580 nm, the CS showed lower reflectance values (*p* < 0.05) than the other samples. At 590 nm was the isosbestic point for the CS, BBM, and BPM samples, while at 600 nm only for CS and BPM. From 600 to 740 nm, there were significant differences (*p* < 0.05) between all the reformulated burgers and the control sample. Thus, BBM and BPM showed lower % reflectance values whilst BOM had % reflectance values higher than the CS. Finally, the NBS coefficient showed that the change in the color between the CS and BBM, BOM, and BPM samples was appreciable. Therefore, adding the different mushroom flours as sodium chloride replacers produces color differences that consumers would identify, considering that color differences (ΔE) higher than three units can be detected by the human eye [29]. 

In the scientific literature, there are contradictory results in reference to the color values when edible mushrooms were used as salt substitutes in hamburgers. In this sense, there are studies that indicate that there are no variations in the parameters L* and a* while coordinate b* was affected [30]. Meanwhile, other studies stated that there were variations in the L* values while b* and a* coordinates were not affected [8]. This will depend on the type of edible mushroom used for the substitution as well as the percentage of such substitution.

**Figure 3:**
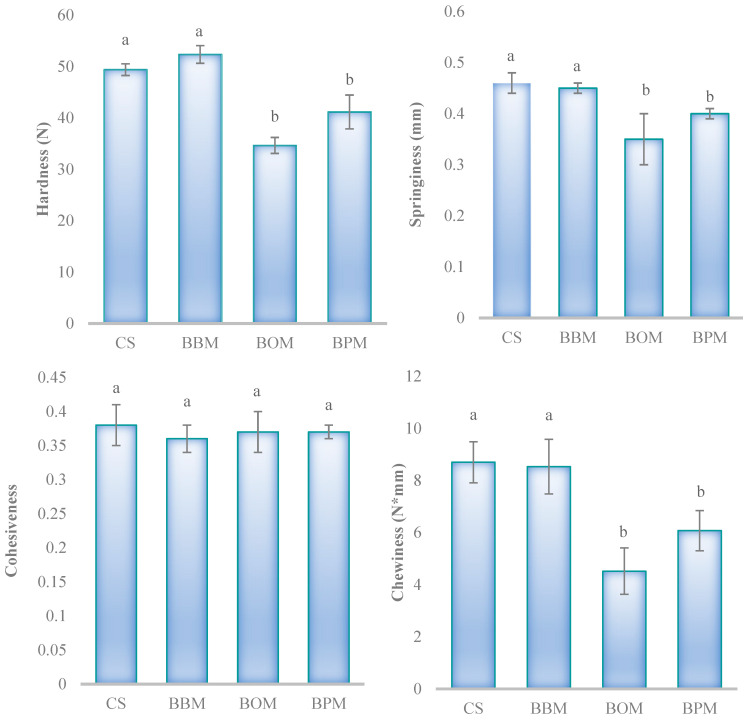
Texture profile analysis of cooked low-sodium chloride beef burgers where oyster mushroom flour, button mushroom flour, and portobello mushroom flour were used as partial sodium chloride replacers. CS: control sample of burgers with a traditional formula; BBM: sample with 75% replacement of sodium chloride with *A. bisporus* flour; BOM: sample with 75% replacement of sodium chloride with *P. ostreatus* flour; BPM: sample with 75% replacement of sodium chloride with *A. brunnescens* flour. For each parameter, histograms followed by the same lowercase letter (a–b are not significantly different according to Tukey’s HSD post-hoc test (*p* > 0.05).

For the texture analysis (Figure 3) of cooked low-sodium chloride beef burgers, only the use of oyster flour and portobello flour as sodium chloride replacers had a significant (*p* < 0.05) effect on hardness, springiness, and chewiness. Thus, BOM and BPM samples showed lower values for these parameters than the control sample, while no statistical differences were found (*p* > 0.05) between the CS and BBM samples for these textural parameters. In the case of cohesiveness, no differences (*p* > 0.05) were obtained between the CS and reformulated samples. These results were in concordance with Patinho et al. [8], who mentioned that beef burgers added with mushrooms as salt replacers showed lower hardness and chewiness compared with the control sample. In a similar study, Cerón-Guevara et al. [15] stated that the hardness, springiness, and chewiness of reformulated beef patties with *A. bisporus* and *P. ostreatus* flours were lower than the control sample. Generally, the addition of several non-meat ingredients, mainly of vegetal origin, in reformulated meat products tends to give softer products when compared to the original.

### 3.3. Cooking Properties

**Figure 4:**
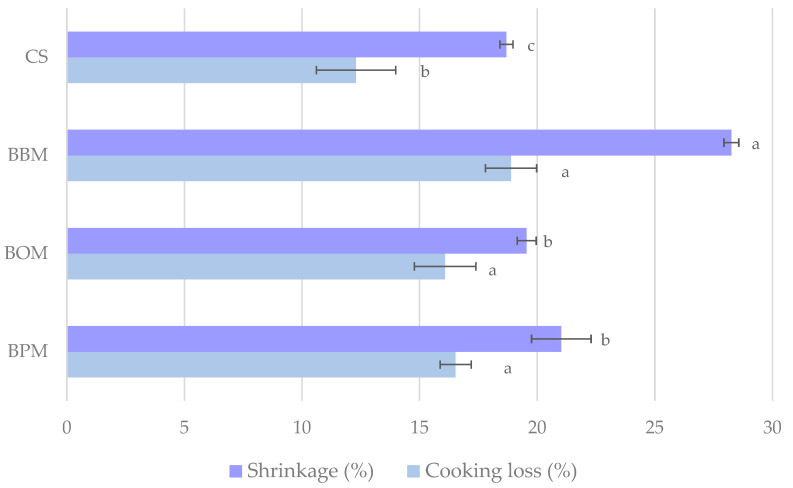
Cooking properties of cooked low-sodium chloride beef burgers where oyster mushroom flour, button mushroom flour, and portobello mushroom flour were used as partial sodium chloride replacers. CS: control sample of burgers with a traditional formula; BBM: sample with 75% replacement of sodium chloride with *A. bisporus* flour; BOM: sample with 75% replacement of sodium chloride with *P. ostreatus* flour; BPM: sample with 75% replacement of sodium chloride with *A. brunnescens* flour. For each parameter, histograms followed by the same lowercase letter (a–c) are not significantly different according to Tukey’s HSD post-hoc test (*p* > 0.05).

In general terms, the sodium chloride reduction in meat products produces a reduction in the water-holding capacity. This fact affects cooking loss which is a very important characteristic of the meat industry because this could lead to yield loss [31]. The cooking loss of samples was significantly affected (*p <* 0.05) by the sodium chloride replacement with the different mushroom flours (Figure 4). Thus, the control sample showed the lowest (*p <* 0.05) cooking loss whereas no statistical differences were found (*p* > 0.05) in BBM, BOM, and BPM samples. These results were in agreement with those reported by Wong, et al., [9] who found that beef patties added with button mushroom (50%) as sodium chloride replacer had higher cooking loss than the control sample. Similarly, Rios-Mera et al., [32] informed that beef burgers with a sodium chloride reduction of 50 and 75% had a cooking loss higher than 39% while the control sample was 33%. These values obtained were expected since sodium chloride is added to meat products and has the property of solubilizing myofibrillar proteins to increase their hydration and water-holding capacity [33]. In this sense, a low sodium chloride concentration affected the heat-induced gelation in meat products as a result of low myofibrillar protein dissolution. On the other hand, an elevated concentration of sodium chloride minimized the extracted water by increasing the solubility and strength of the myofibrillar proteins network [34]. Thus, sodium chloride concentrations lower than 1.0% increase the cooking loss of meat products, mainly due to water loss [35]. 

Avoiding shrinkage is considered an important issue to maintain the quality of the burgers due to consumers associating a higher degree of shrinkage with poor meat quality, the addition of a large amount of water to the product, and/or the effect of hormone treatments. As occurs with cooking loss, the shrinkage of samples was significantly affected (*p* < 0.05) by the sodium chloride replacement with the different mushroom flours. As shown in Figure 4, the BBM, BOM, and BPM samples had higher (*p* < 0.05) shrinkage values than the control sample. Between BOM and BPM, no statistical differences were found (*p* > 0.05), while BBM had the highest (*p* < 0.05) value. Similar results were obtained by Mattar et al. [30], who reported that beef burgers with a 50% reduction of sodium chloride and the addition of mushroom flour had shrinkage values higher than the control sample. As mentioned by Besbes et al. [36], shrinkage is the result of the denaturation of meat proteins with the loss of water and fat; the reduction of salt content might contribute to the increase of this phenomenon due to the capacity of salt to solubilize proteins and improve the water holding capacity of their water and fat-binding capacities. Therefore, the lower the amount of salt in the product, the lower the water holding capacity and the higher the % shrinkage.

### 3.4. Lipid Oxidation 

**Figure 5:**
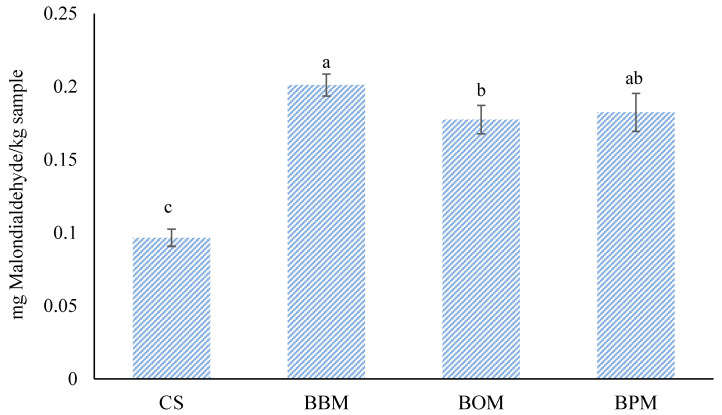
Lipid oxidation values of cooked low-sodium chloride beef burgers where oyster mushroom flour, button mushroom flour, and portobello mushroom flour were used as partial (75%) sodium chloride replacers. CS: control sample of burgers with a traditional formula; BBM: sample with 75% replacement of sodium chloride with *A. bisporus* flour; BOM: sample with 75% replacement of sodium chloride with *P. ostreatus* flour; BPM: sample with 75% replacement of sodium chloride with *A. brunnescens* flour. Histograms followed by the same lowercase letter (a–c) are not significantly different according to Tukey’s HSD post-hoc test (*p* > 0.05).

The effect on the oxidative stability of low-sodium chloride beef burgers where oyster mushroom flour, button mushroom flour, and portobello mushroom flour were used as partial (75%) sodium chloride replacers was determined by measuring the thiobarbituric acid reacting substances (TBARS) of cooked samples, and the results are presented in Figure 5. A significant (*p* < 0.05) increase in the TBARS values was observed in burgers reformulated with different edible mushroom flours compared to the control burgers, obtaining the highest value (*p* < 0.05) when button mushroom (0.2 mg MDA/kg) was incorporated as a sodium chloride replacer. Overall, as can be seen in Figure 5, the TBARS values of all samples ranged from 0.10 to 0.21 mg MDA/kg, being below the threshold level of 0.6 mg MDA/kg above which undesirable sensory changes related to rancid flavors may be perceived by consumers [37].

This increase in the TBARS values has been previously observed by Cerón-Guevara et al. [15] when they formulated beef patties with *A. bisporus* and *P. ostreatus* flours. These authors reported that this increase in the TBARS values caused by the low antioxidant activity of mushroom flours could be related to the drying process applied to obtain the flours, which can cause damage to the antioxidant compounds. Contrary to this study, Patinho et al. [8] reported that the TBARS values of beef burgers added with button mushroom flour as sodium chloride replacers were lower than those obtained for the control sample. Similarly, Alnoumani et al. [38] reported that the reduction in the TBARS values of ground beef added with button mushroom flour (1%) is related to a synergistic effect between the sodium chloride content and the mushroom flour. On the other hand, Pachekrepapol et al. [39] found that the TBARS values of catfish and salmon burgers added with oyster mushrooms were not significantly different compared with the control sample. 

### 3.5. Sensorial Analysis

Table 4 shows the sensory analysis of cooked low-sodium chloride beef burgers where oyster mushroom flour, button mushroom flour, and portobello mushroom flour were used as partial (75%) sodium chloride replacers. All the parameters analyzed were not affected (*p* > 0.05) by the replacement of sodium chloride by the different mushroom flours. The hedonic scores for all burgers analyzed, with a few exceptions, ranged from 6 to 7. It is important to note that for color, despite the raw samples presenting a notable difference in the appreciation of the redness coordinate (a*) and the redness index between the BBM, BOM, and BPM samples and the CS, this was not appreciated by the consumers after the cooking process, and these samples were even better valued than the CS, although without significant differences. Something similar happened with the rancidity since differences were detected instrumentally between the control sample, which showed a lower degree of oxidation, and the samples added with the different edible mushroom flours. However, this circumstance was not detected by the consumers in the analysis performed. It is also important to highlight that although hardness values were in the CS and BBM sample, consumers rated these two samples with the highest scores, which indicates that this is an aspect to be improved in this type of product, which associate high hardness values with higher quality. As for the salty taste, consumers did not detect the 75% sodium chloride reduction in the samples added with the different mushroom flour. Both the control sample and the reformulated samples showed very similar values, which implies that the replacement of sodium chloride by edible mushrooms does not impair this flavor.

## 4. Conclusions

The results found in this study indicate that the use of flours obtained from oyster mushrooms (*Pleurotus ostreatus*), button mushrooms (*Agaricus bisporus*), and portobello mushrooms (*Agaricus brunnescen*) are a viable alternative to be used as sodium chloride replacers in the manufacture of beef burgers. Despite having certain undesirable effects on cooking properties, texture, and color, the final product is not negatively valued by consumers, and the benefits provided, such as the reduction in sodium content between 55 and 61%, are of great interest from a health-related point of view. Moreover, the inclusion of this type of flour would not cause a substantial increase in the final price of the product as it is a raw material that does not have a very high economic value. In the future, it would be important to analyze, from a toxicological point of view, the benefits or disadvantages of the use of mushroom extracts or mushroom flours as salt replacers in meat products.

## Figures and Tables

**Table 1:** Formulation of low-sodium chloride beef burgers where oyster mushroom flour (OM), button mushroom flour (BM), and portobello mushroom flour (PM) were used as partial (75%) sodium chloride replacers.

	CS	BOM	BBM	BPM
Beef	78	78	78	78
Pork backfat	15	15	15	15
Water	5	5	5	5
Sodium chloride	1.8	0.45	0.45	0.45
Black pepper	0.2	0.2	0.2	0.2
OM	0	1.35	0	0
BM	0	0	1.35	0
PM	0	0	0	1.35

Values expressed as g/100 g.

**Table 2:** Chemical composition of raw and cooked low-sodium chloride beef burgers where oyster mushroom flour, button mushroom flour, and portobello mushroom flour were used as partial (75%) sodium chloride replacers.

	RAW				COOKED			
	CS	BBM	BOM	BPM	CS	BBM	BOM	BPM
**Moisture**	64.37 ± 0.72 ^a^	66.87 ± 0.48 ^a^	65.17 ± 1.73 ^a^	64.94 ± 1.83 ^a^	61.30 ± 0.08 ^a^	59.55 ± 0.29 ^b^	59.83 ± 0.16 ^b^	59.45 ± 0.46 ^b^
**Protein**	15.04 ± 0.14 ^c^	15.72 ± 0.05 ^b^	15.61 ± 0.23 ^b^	16.57 ± 0.37 ^a^	17.44 ± 0.15 ^b^	19.55 ± 0.48 ^a^	18.72 ± 0.33 ^a^	19.36 ± 0.09 ^a^
**Fat**	12.67 ± 0.50 ^a^	12.51 ± 0.27 ^a^	12.38 ± 0.67 ^b^	12.13 ± 0.52 ^a^	13.69 ± 0.35 ^a^	13.13 ± 0.22 ^a^	13.22 ± 0.11 ^a^	13.26 ± 0.25 ^a^
**Ash**	2.52 ± 0.10 ^a^	1.53 ± 0.01 ^b^	1.43 ± 0.02 ^b^	1.52 ± 0.10 ^b^	2.89 ± 0.09 ^a^	1.80 ± 0.12 ^b^	1.69 ± 0.11 ^b^	1.67 ± 0.05 ^b^
**Sodium**	824.25 ± 18.94 ^a^	302.22 ± 0.54 ^c^	372.25 ± 17.69 ^b^	361.13 ± 7.37 ^b^	1026.16 ± 16.78 ^a^	459.60 ± 6.06 ^b^	462.01 ± 26.12 ^b^	400.61 ± 5.75 ^c^
**Potassium**	169.79 ± 3.19 ^c^	223.66 ± 4.75 ^b^	224.64 ± 1.99 ^b^	235.58 ± 6.43^a^	184.20 ± 3.46 ^c^	283.89 ± 2.65 ^a^	271.52 ± 4.54 ^b^	264.64 ± 4.70 ^b^
**Calcium**	24.27 ± 0.22 ^a^	21.87 ± 0.53 ^b^	16.91 ± 0.20 ^d^	18.17 ± 0.57 ^c^	22.31 ± 0.17 ^b^	23.10 ± 0.11 ^a^	20.44 ± 0.43 ^c^	23.13 ± 0.31 ^a^
**Cupper**	Traces	Traces	Traces	Traces	Traces	Traces	Traces	Traces
**Iron**	1.46 ± 0.03 ^d^	1.56 ± 0.03 ^c^	1.69 ± 0.02 ^a^	1.62 ± 0.02 ^b^	1.70 ± 0.05 ^b^	1.55 ± 0.02 ^c^	1.97 ± 0.13 ^a^	1.77 ± 0.05 ^b^
**Magnesium**	19.57 ± 0.29 ^c^	21.33 ± 0.24 ^b^	22.67 ± 0.15 ^a^	22.23 ± 0.24 ^a^	21.83 ± 0.52 ^d^	26.95 ± 0.46 ^b^	29.25 ± 1.23 ^a^	24.89 ± 0.65 ^c^
**Manganese**	Traces	Traces	Traces	Traces	Traces	Traces	Traces	Traces
**Zinc**	3.07 ± 0.04 ^c^	3.39 ± 0.06 ^a^	3.22 ± 0.02 ^b^	3.02 ± 0.05 ^c^	2.92 ± 0.09 ^c^	3.36 ± 0.03 ^a^	3.12 ± 0.05 ^b^	3.15 ± 0.05 ^b^

Results for moisture protein, fat, and ash were expressed as g/100 g. Mineral content was expressed as mg/100 g. CS: control sample of burgers with a traditional formula; BBM: sample with 75% of replacement of sodium chloride with *A. bisporus* flour; BOM: sample with 75% of replacement of sodium chloride with *P. ostreatus* flour; BPM: sample with 75% of replacement of sodium chloride with *A. brunnescens* flour. For each parameter, results followed by the same lowercase letter (a–c) are not significantly different according to Tukey’s HSD post-hoc test (*p* > 0.05). Traces = <1 mg/100 g.

**Table 3:** Physicochemical properties of raw low-sodium chloride beef burgers where oyster mushroom flour, button mushroom flour, and portobello mushroom flour were used as partial (75%) sodium chloride replacers.

Sample	pH	Aw	L*	a*	b*	RI	NBS
**CS**	5.67 ± 0.03 ^b^	0.964 ± 0.00 ^b^	47.47 ± 3.13 ^a^	13.53 ± 2.53 ^a^	14.91 ± 1.94 ^a^	0.91 ± 0.15 ^a^	-
**BBM**	5.73 ± 0.01 ^a^	0.975 ± 0.00 ^a^	46.93 ± 2.37 ^a^	8.35 ± 0.98 ^b^	13.56 ± 1.57 ^a^	0.62 ± 0.05 ^b^	4.95 ± 0.84 ^a^
**BOM**	5.66 ± 0.03 ^b^	0.976 ± 0.00 ^a^	50.18 ± 3.65 ^a^	9.48 ± 3.40 ^b^	14.75 ± 2.77 ^a^	0.63 ± 0.09 ^b^	4.48 ± 0.63 ^a^
**BPM**	5.73 ± 0.01 ^a^	0.976 ± 0.00 ^a^	47.81 ± 2.28 ^a^	9.24 ± 2.22 ^b^	15.32 ± 1.73 ^a^	0.60 ± 0.12 ^b^	4.01 ± 0.69 ^a^

CS: control sample of burgers with a traditional formula; BBM: sample with 75% replacement of sodium with *A. bisporus* flour; BOM: sample with 75% replacement of sodium with *P. ostreatus* flour; BPM: sample with 75% replacement of sodium with *A. brunnescens* flour. For each parameter, results followed by the same lowercase letter (a, b) are not significantly different according to Tukey’s HSD post-hoc test (*p* > 0.05).

**Table 4:** Sensory analysis of cooked raw low-sodium chloride beef burgers where oyster mushroom flour, button mushroom flour, and portobello mushroom flour were used as partial sodium chloride replacers.

Sample	Visual Aspect	Color	Hardness	Flavor	Salty Taste	Rancidity	Acceptability
**CS**	5.67 ± 0.03 ^b^	0.964 ± 0.00 ^b^	47.47 ± 3.13 ^a^	13.53 ± 2.53 ^a^	14.91 ± 1.94 ^a^	0.91 ± 0.15 ^a^	-
**BBM**	5.73 ± 0.01 ^a^	0.975 ± 0.00 ^a^	46.93 ± 2.37 ^a^	8.35 ± 0.98 ^b^	13.56 ± 1.57 ^a^	0.62 ± 0.05 ^b^	4.95 ± 0.84 ^a^
**BOM**	5.66 ± 0.03 ^b^	0.976 ± 0.00 ^a^	50.18 ± 3.65 ^a^	9.48 ± 3.40 ^b^	14.75 ± 2.77 ^a^	0.63 ± 0.09 ^b^	4.48 ± 0.63 ^a^
**BPM**	5.73 ± 0.01 ^a^	0.976 ± 0.00 ^a^	47.81 ± 2.28 ^a^	9.24 ± 2.22 ^b^	15.32 ± 1.73 ^a^	0.60 ± 0.12 ^b^	4.01 ± 0.69 ^a^

CS: control sample of burgers with a traditional formula; BBM: sample with 75% replacement of sodium with *A. bisporus* flour; BOM: sample with 75% replacement of sodium with *P. ostreatus* flour; BPM: sample with 75% replacement of sodium with *A. brunnescens* flour. For each parameter, results followed by the same lowercase letter (a, b) are not significantly different according to Tukey’s HSD post-hoc test (*p* > 0.05).

## Data Availability

Data are available upon request to the authors.

## Supplementary Materials

The following are available online at https://www.mdpi.com/article/10.3390/foods12193591/s1, Figure S1. Reflectance spectra (360–740 nm) of raw low-sodium chloride beef burgers where oyster mushroom flour, button mushroom flour, and portobello mushroom flour were used as partial (75%) sodium chloride replacers.
