# Supplementary material for: Assessment of Chemical, Physico-Chemical and Sensory Properties of Low-Sodium Beef Burgers Formulated with Flours from Different Mushroom Types
Source: Foods. 2023 Sep 27;12(19):3591. doi: 10.3390/foods12193591 (PMC10572391; doi:10.3390/foods12193591)
Supplement: Supplementary file 1 [file foods-12-03591-s001.zip › foods-2633937-supplementary.pdf]

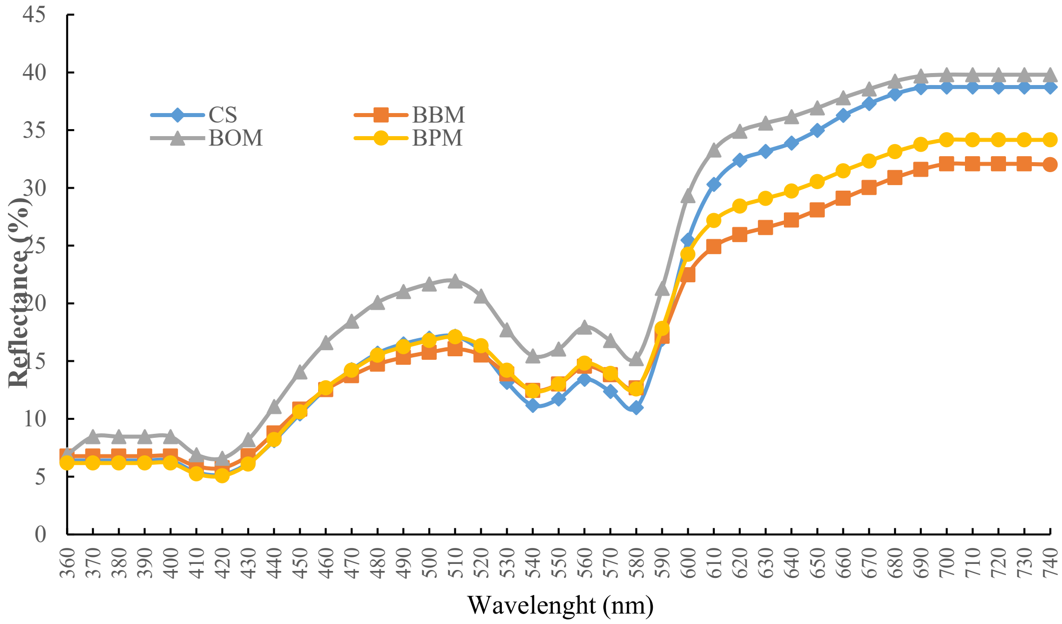

Figure S1 . Reflectance spectra (360-740 nm) of raw low-sodium chloride beef burgers where oyster mushroom flour, button mushroom flour and portobello mushroom flour were used as partial (75%) sodium chloride replacers.
